# Supplementary material for: Toward an integrated map of genetic interactions in cancer cells
Source: Mol Syst Biol. 2018 Feb 21;14(2):e7656. doi: 10.15252/msb.20177656 (PMC5820685; doi:10.15252/msb.20177656)
Supplement: Supplementary file 1 — Expanded View Figures PDF [file MSB-14-e7656-s001.pdf]

# Expanded View Figures

**Figure EV1. Integration and normalization of viability phenotypes in different screens.**

A Schematic workflow of the normalization process.

B Schematic illustration of the gene-level batch adjustment to correct for differences across libraries. Each box in the plot corresponds to the phenotype observed in screens using the same sgRNA library when targeting a specific gene of interest. In case the phenotypes of one library differ significantly (FDR < 5%; Benjamini–Hochberg) from the expected (median of all phenotypes for the same gene), the library is adjusted by subtracting the estimated difference between the mean of the library and the expected phenotype from each data point corresponding to that library.

C Examples of two genes where batch effects are observed showing the effects of the normalization.

D Example where an sgRNA library associated batch effect can, if not adjusted, lead to a false-positive interaction between PSMA1 and RNF43. Each dot represents one screening experiment. The RNF43 mutated group is comprised of 14 data points, the RNF43 wt group consists of 71 data points.

E Four examples of genetic interactions reported previously where phenotypes are conserved through the normalization process. Each dot represents one screening experiment. The number of data points corresponding to each group are 7 (FZD5 konkcout, RNF43 mut.), 12 (FZD5 knockout, RNF43 wt), 9 (all RAS wt groups) and 5 (all RAS mut. groups). Horizontal red lines indicate the group means.

F Clustering of normalized CRISPR scores of all experiments used in the analysis based on genes shared across libraries. The heat map is similar to the one shown in Fig 1A with all columns labeled.

Data information: (B–D) Red dotted lines indicate the median CRISPR score across all libraries. Horizontal lines inside each box indicate the median. The upper and lower boundaries of each box represent the 75$^{th}$ and 25$^{th}$ percentile, respectively. Error bars indicate the 10$^{th}$ and 90$^{th}$ percentiles and outliers are depicted as circles.


© 2018 The Authors

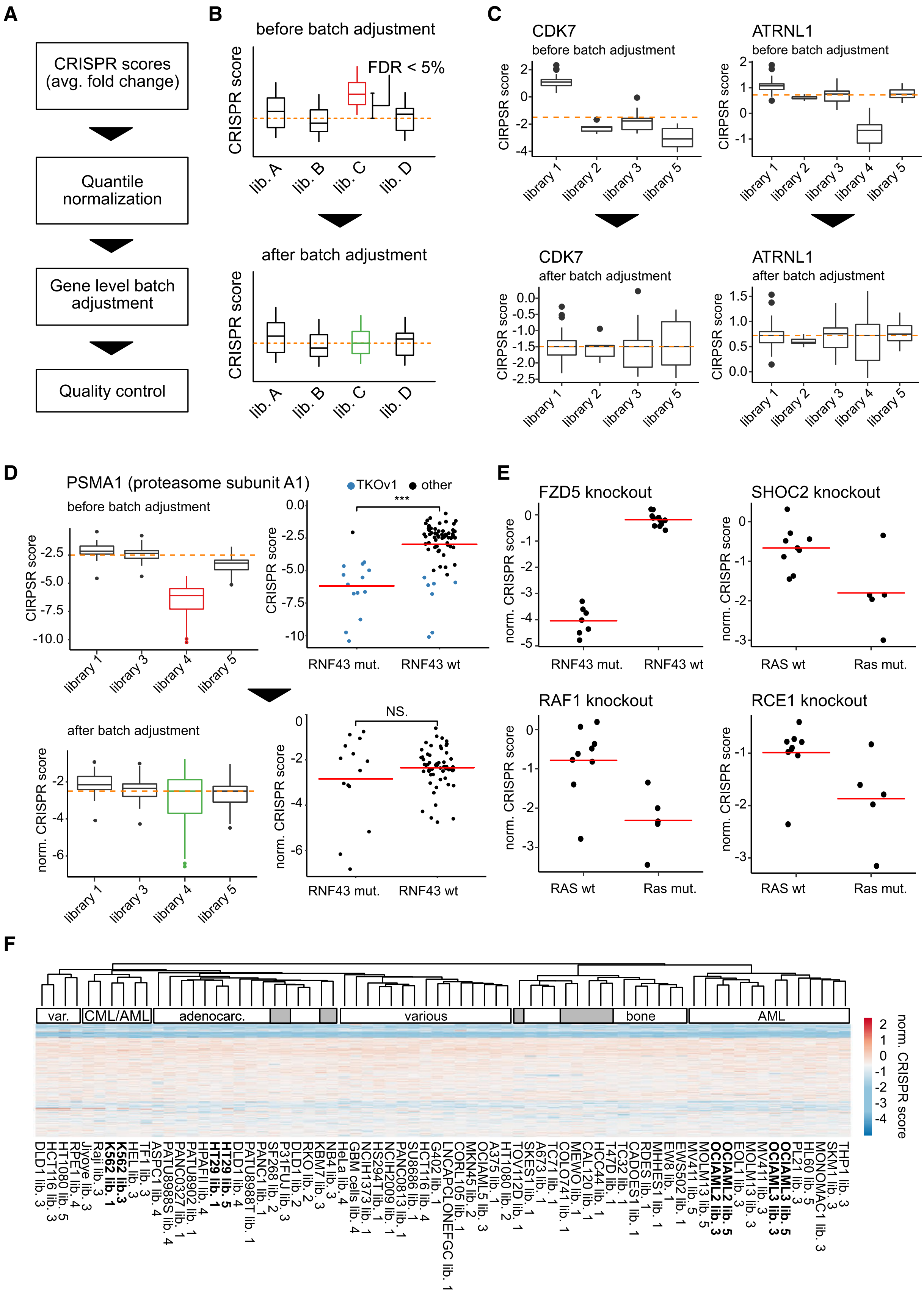
A
CRISPR scores (avg. fold change)
Quantile normalization
Gene level batch adjustment
Quality control
B
before batch adjustment
FDR < 5%
CRISPR score
lib. A
lib. B
lib. C
lib. D
after batch adjustment
C
CDK7
before batch adjustment
CIRPSR score
library 1
library 2
library 3
library 5
ATRNL1
before batch adjustment
library 4
CDK7
after batch adjustment
ATRNL1
after batch adjustment
D
PSMA1 (proteasome subunit A1)
before batch adjustment
TKOv1
other
***
CIRPSR score
CRISPR score
RNF43 mut.
RNF43 wt
after batch adjustment
NS.
norm. CRISPR score
E
FZD5 knockout
SHOC2 knockout
RAS wt
Ras mut.
RAF1 knockout
RCE1 knockout
F
var.
CML/AML
adenocarc.
various
bone
AML
norm. CRISPR score
DLD1 lib. 3
HCT116 lib. 3
HT1080 lib. 5
RPE1 lib. 4
Jiyoye lib. 3
Raji lib. 3
K562 lib. 1
K562 lib. 3
HEL lib. 3
TF1 lib. 3
ASPC1 lib. 4
PATU8988S lib. 4
PANC0327 lib. 1
PATU8902 lib. 1
HPAFII lib. 4
HT29 lib. 1
HT29 lib. 5
DLD1 lib. 4
PATU8988T lib. 1
PANC1 lib. 1
SF268 lib. 2
P31FUJ lib. 3
DLD1 lib. 2
RKO lib. 2
KBM7 lib. 3
NB4 lib. 3
HeLa lib. 4
GBM cells lib. 4
NCIH1373 lib. 1
HS294T lib. 1
NCIH2009 lib. 1
PANC0813 lib. 1
SU8686 lib. 1
HCT116 lib. 4
G402 lib. 1
LNCAPCLONEFGC lib. 1
CORL105 lib. 1
MKN45 lib. 2
OCIAML5 lib. 3
A375 lib. 1
HT1080 lib. 2
TOV112D lib. 1
SKES1 lib. 1
A673 lib. 1
TC71 lib. 1
COLO741 lib. 1
MEWO lib. 1
CAL120 lib. 1
HCC44 lib. 1
T47D lib. 1
TC32 lib. 1
CADOES1 lib. 1
RDES lib. 1
MHHES1 lib. 1
EW8 lib. 1
EWS502 lib. 1
MV411 lib. 5
MOLM13 lib. 5
OCIAML2 lib. 3
OCIAML2 lib. 5
EOL1 lib. 3
MOLM13 lib. 3
MV411 lib. 3
OCIAML3 lib. 3
OCIAML3 lib. 5
PL21 lib. 3
HL60 lib. 5
MONOMAC1 lib. 3
SKM1 lib. 3
THP1 lib. 3

**Figure EV1.**

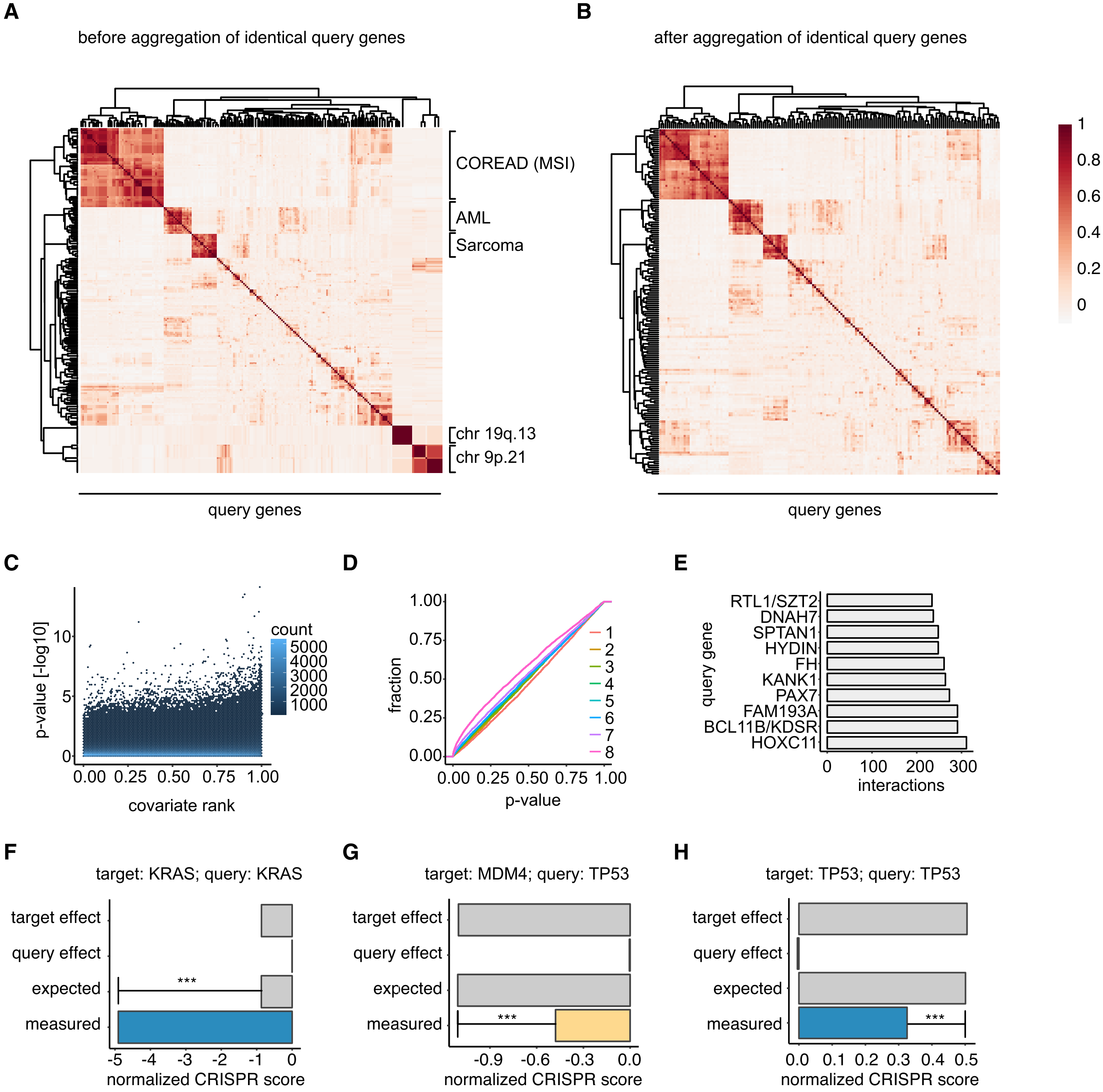


**Figure EV2. Prediction of genetic interactions between gene pairs.**

- A Pairwise correlations between query genes. Clusters of highly correlating query genes are labeled indicating the source of the correlation.
- B Query correlations after aggregation of fully (Pearson's correlation coefficient = 1) correlated query genes.
- C Scatter plot of log10-scaled *P*-values against the covariate rank of each gene pair as used for multiple testing correction using IHW. The covariate is the variance of CRISPR scores of the group of mutated cell lines.
- D Empirical cumulative distribution function plot of *P*-values of genetic interactions based on the covariate.
- E Ten query genes with the highest predicted interaction count. Multiple gene symbols separated by forward slashes indicate query genes that were aggregated due to high correlation.
- F–H Epistasis plots for three examples of known genetic interactions. Blue color indicates a negative interaction, and yellow indicates a positive interaction. To determine a genetic interaction, the difference between the measured and the expected combined phenotype is quantified. Statistical significance was determined using a linear mixed effects model with the lmerTest method. ***, FDR < 0.0001.


© 2018 The Authors

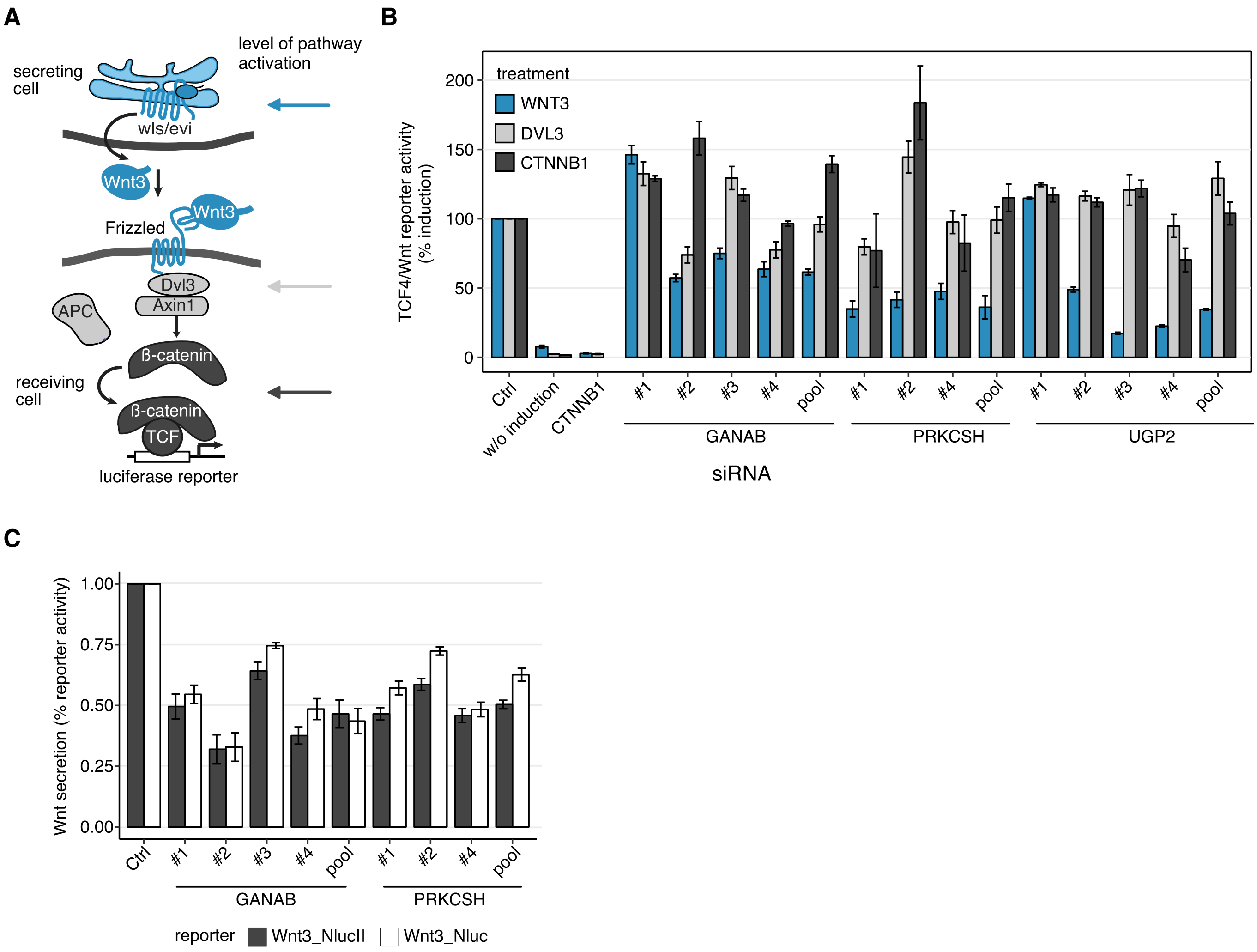


**Figure EV3. Analysis of candidate regulators of Wnt/β-catenin signaling.**

A Schematic explaining the Wnt/TCF4 reporter assay used to determine the influence of candidate genes on Wnt/β-catenin signaling. Arrows indicate levels of pathway activation by overexpression of WNT3 (blue), DVL3 (gray), or CTNNB1/β-catenin (black).

B Complete data for the Wnt activity essay presented in Fig 4B. Groups of bars indicate the effects of individual siRNAs targeting the candidate genes. Results are shown as averages of 3–4 independent experiments ± s.e.m.

C Complete data for Wnt secretion essay presented in Fig 4C. Groups of bars illustrate the effect of individual siRNA perturbations. Results are shown as averages of three independent experiments ± s.e.m.

© 2018 The Authors

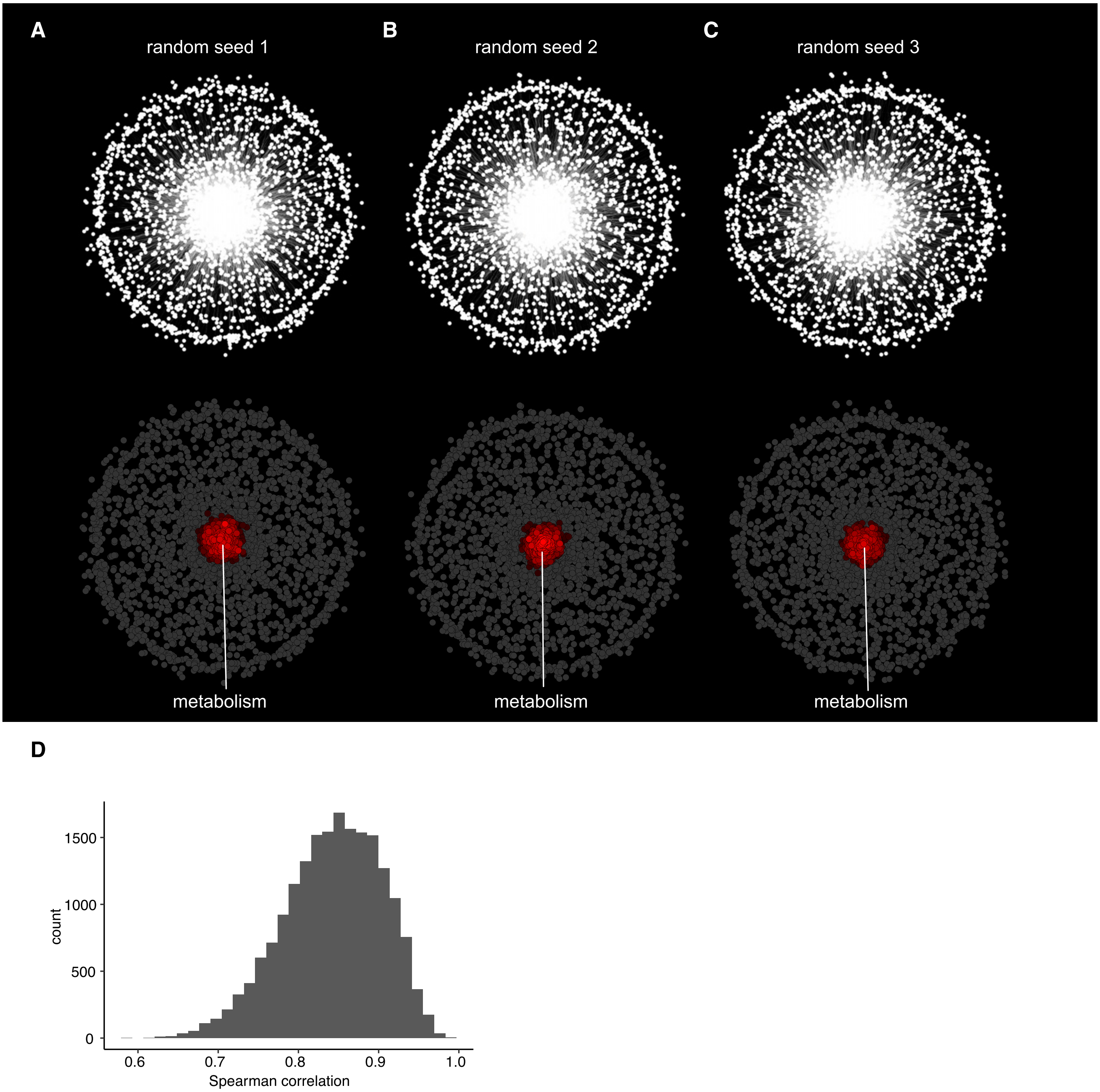


**Figure EV4. Computational validation of the predicted genetic network.**

A–C Genetic network after random permutation of edges using three different random seeds. A force-directed spring-embedded layout was applied to position nodes similar to the network shown in Fig 5. Each dot corresponds to a gene. Genes in the network as well as the number of edges were maintained.

D Histogram of Spearman's correlation coefficients representing the edges of the genetic network shown in Fig 5D.


© 2018 The Authors
